# SIRT1 Activators Suppress Inflammatory Responses through Promotion of p65 Deacetylation and Inhibition of NF-κB Activity

**DOI:** 10.1371/journal.pone.0046364

**Published:** 2012-09-28

**Authors:** Hongying Yang, Wei Zhang, Heng Pan, Heidi G. Feldser, Elden Lainez, Christine Miller, Stewart Leung, Zhong Zhong, Huizhen Zhao, Sharon Sweitzer, Thomas Considine, Thomas Riera, Vipin Suri, Brian White, James L. Ellis, George P. Vlasuk, Christine Loh

**Affiliations:** 1 Sirtris, a GSK Company, Cambridge, Massachusetts, United States of America; 2 GlaxoSmithKline R&D China, Shanghai, People’s Republic of China; 3 GlaxoSmithKline, Collegeville, Pennsylvania, United States of America; French National Centre for Scientific Research, France

## Abstract

Chronic inflammation is a major contributing factor in the pathogenesis of many age-associated diseases. One central protein that regulates inflammation is NF-κB, the activity of which is modulated by post-translational modifications as well as by association with co-activator and co-repressor proteins. SIRT1, an NAD^+^-dependent protein deacetylase, has been shown to suppress NF-κB signaling through deacetylation of the p65 subunit of NF-κB resulting in the reduction of the inflammatory responses mediated by this transcription factor. The role of SIRT1 in the regulation of NF-κB provides the necessary validation for the development of pharmacological strategies for activating SIRT1 as an approach for the development of a new class of anti-inflammatory therapeutics. We report herein the development of a quantitative assay to assess compound effects on acetylated p65 protein in the cell. We demonstrate that small molecule activators of SIRT1 (STACs) enhance deacetylation of cellular p65 protein, which results in the suppression of TNFα-induced NF-κB transcriptional activation and reduction of LPS-stimulated TNFα secretion in a SIRT1-dependent manner. In an acute mouse model of LPS-induced inflammation, the STAC SRTCX1003 decreased the production of the proinflammatory cytokines TNFα and IL-12. Our studies indicate that increasing SIRT1-mediated NF-κB deacetylation using small molecule activating compounds is a novel approach to the development of a new class of therapeutic anti-inflammatory agents.

## Introduction

Inflammation is a physiological response to remove injurious stimuli and initiate the healing process. However, unresolved or sustained low-grade inflammation leads to development of chronic diseases including chronic obstructive pulmonary disease (COPD), rheumatoid arthritis, type 2 diabetes (T2D), cancer, Alzheimer’s disease, cardiovascular, and renal diseases, many of which are associated with aging. Upregulation of inflammatory biomarkers is a characteristic of the aging process [1]. Thus, inflammation is considered a major contributing factor in the pathogenesis of many age-related diseases [1].

One key protein that regulates inflammatory responses is the transcription factor NF-κB which is held quiescent in the cytoplasm when in complex with IκBα. In response to a proinflammatory stimulus (e.g. lipopolysaccharide (LPS), tumor necrosis factor (TNFα), or interleukin-1 (IL-1)) via Toll-like receptors or cytokine receptors, IκBα is phosphorylated by IKK and subject to ubiquitin-dependent proteasomal degradation, thereby allowing NF-κB to translocate to the nucleus and activate the transcription of a cascade of proinflammatory cytokines and chemokines to induce inflammatory responses [2], [3]. Activation of NF-κB-regulated gene expression is also modulated by post-transcriptional modifications, such as phosphorylation, acetylation and methylation, which can be altered upon stimulation [3], [4], [5], [6]. Of particular interest is the acetylation of p65/RelA, a subunit of the heterodimeric NF-κB protein, which can either potentiate or diminish NF-κB signaling depending on the particular acetylated lysine residue [7], [8]. Among the seven lysines (lysine 122, 123, 218, 221, 310, 314, 315) that are acetylated by p300/CBP and PCAF [7], [8], [9], [10], [11], [12], [13], [14], acetylation of lysine 310 is critical for full activation of NF-κB transcription potential [7], which can be deacetylated by SIRT1 [15].

SIRT1 is an NAD^+^-dependent protein deacetylase that plays important roles in regulating metabolism, inflammation, stress resistance, DNA repair and cell survival through deacetylation of key transcription factors, enzymes and proteins [16], [17]. Following the initial report by Yeung *et al.* that SIRT1 can deacetylate p65 at lysine 310 [15], other studies have also demonstrated the inhibitory effect of SIRT1 on NF-κB-mediated inflammation. Overexpression of SIRT1 or activation of SIRT1 by resveratrol (RES) promotes deacetylation of p65 and suppression of transcriptional activation by NF-κB, resulting in protection against microglia-dependent amyloid-β toxicity in neurons [18]. SIRT1 protein is decreased in the lungs of rats exposed to cigarette smoke as well as in lungs of smokers and patients with COPD [19], [20]. Increasing SIRT1 activity by gene overexpression or pharmacological activation by RES inhibits, whereas decreasing SIRT1 activity by gene knockdown or inhibition of SIRT1 by sirtinol potentiates inflammatory responses, presumably via SIRT1-mediated deacetylation of p65 [19], [20]. Further, transgenic mice with modest SIRT1 overexpression on high fat diet (HFD) show reduced levels of proinflammatory cytokines such as IL-6 and TNFα [21]. Conversely, myeloid cell-specific SIRT1 knockout mice show increased secretion of these cytokines when challenged with LPS, and are predisposed to the development of systemic insulin resistance and metabolic disorders upon HFD feeding [22].

The pivotal role of SIRT1 in regulating inflammation suggests a new avenue for attenuating inflammation by modulating SIRT1 activity. SIRT1 activity can be regulated by the endogenous activator AROS (active regulator of SIRT1) [23], inhibitors (deleted in breast cancer-1 (DBC1) and Tat) [24], [25], [26], NAD^+^ concentration [27], and by posttranslational modifications such as phosphorylation [28], [29], [30]. Deletion of DBC1 results in increased SIRT1 activity and renders mice resistant to HFD-induced liver steatosis and inflammation [31]. Likewise, administration of the NAD^+^ biosynthesis substrate NMN, which increases NAD^+^ levels, restores HFD-induced p65 hyperacetylation and gene expression related to inflammatory response, leading to improved hepatic insulin sensitivity [32]. In addition to endogenous regulation of SIRT1, direct pharmacological modulators of SIRT1 activity have also been reported [33].

Resveratrol (RES) was first identified as a naturally occurring small molecule that biochemically activates SIRT1 [34]. While RES exhibits anti-inflammatory effects [15], [18], [19], it is pharmacologically complex and likely involves additional targets depending upon the dose used [35], [36]. To more directly understand the role of SIRT1 in inflammation, we have identified novel synthetic SIRT1-activating compounds (STACs) chemically distinct from RES. Early examples of STACs have shown potential *in vivo* anti-inflammatory effects in the context of metabolic disease, where cytokines and chronic inflammation have been shown to contribute to the metabolic dysfunction in animal models and cell culture systems [33], [37], [38], [39], [40], [41].

We are interested in understanding the mechanism by which STACs regulate inflammation. We therefore developed a quantitative assay to measure the cellular levels of acetylated p65 protein. In this cell-based system we demonstrate that overexpression of SIRT1 attenuates, while knockdown or inhibition of SIRT1 increases p65 acetylation. We also show that compounds from two chemical series of STACs activate SIRT1 *in vitro* and promote SIRT1-mediated deacetylation of p65 protein in cells. Furthermore, these STACs can attenuate p65 acetylation and inhibit NF-κB activation induced by TNFα and blunt LPS-stimulated TNFα secretion. Finally, we show STAC SRTCX1003 mediates decreases in the production of several proinflammatory cytokines, including TNFα and IL-12, *in vivo* following the administration of LPS. Our studies indicate that small molecule activators of SIRT1 are potential drug development candidates for treating inflammation through downregulation of NF-κB signaling.

## Materials and Methods

### Structures and Synthesis of STACs

**Figure 1:**
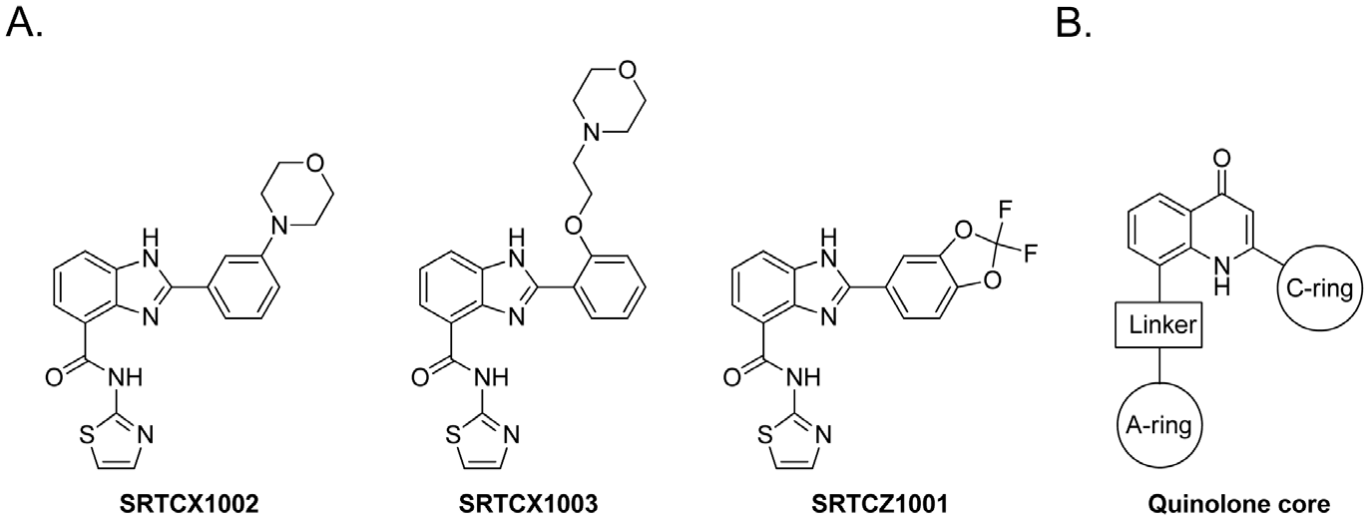
Structures of STACs. (A) Structures of the benzimidazole STACs. (B) Core structure of the quinolone STACs.

Structures of benzimidazole STACs and the core structure of quinolone STACs of this study are shown in Figure 1. Benzimidazole STACs [42], [43] and quinolone STACs [44] were prepared according to the procedures described in the relevant patents.

### Cell Culture and Reagents

U2OS cells (ATCC, HTB-96) and HEK 293 cells (cell line used for BacMam transduction was derived at GSK from ATCC CRL-1573 [45]) were cultured in DMEM/F12 (Invitrogen, 10565-018) with 10% Fetal Bovine Serum (FBS) (Gibco). HEK 293T/17 (ATCC, CRL-11268) cells, RAW 264.7 macrophages (ATCC #TIB-71) and HEK 293 cells (ATCC, CRL-1573) stably expressing NF-κB luciferase reporter were maintained in DMEM (Invitrogen #11995) with 10% FBS (Gibco). EX-527 [46] was synthesized at Sirtris.

### Quantitative Assay for Measuring Cellular Acetylated p65 Levels

BacMam p65 and BacMam p300-HAT viruses were prepared internally according to the standard procedures [47] and were transduced into U2OS cells or HEK 293 cells at 2% and 1% (vol/vol). Cell suspensions containing viruses were then plated into 384-well plates by Multidrop Dispenser. Six hours later, test compounds were added into each well by Apricot liquid handler. Acetylated p65 protein in cell lysates was measured at 24 hours post viral transduction. For the experiments to test SIRT1 overexpression on p65 acetylation, BacMam SIRT1 or BacMam GFP virus was added into cells immediately after BacMam p65 and BacMam p300 viruses were transduced. For the experiments to evaluate knockdown of SIRT1 on p65 acetylation, SIRT1-siRNA or NT-siRNA was transfected into cells 24 hours prior to transduction of BacMam p65 and BacMam p300 viruses, which allowed 48 hours for SIRT1-siRNA to induce significant knockdown of SIRT1.

Acetylated p65 protein in cell lysates was measured by an AlphaScreen format based assay (Bosse *et al.,* Principles of AlphaScreen. PerkinElmer Application Note (2001)). A sandwich comprised of a biotinylated anti-HA antibody (Roche, 12158167001) bound to streptavidin coated donor beads (PerkinElmer, 6760617M) and an anti-acetylated K310-p65 antibody (Abcam, ab19870) interacting with protein A coated acceptor beads was assembled to detect light emission upon acetylated HA-p65 protein binding to the donor and acceptor beads. A similar AlphaScreen assay to measure total HA-tagged p65 protein was also developed by replacing the anti-acetylated K310-p65 antibody with an anti-p65 antibody (Santa Cruz, sc-109). The level of acetylated p65 protein was normalized against the total HA-tagged p65 protein measured in parallel for the same sample.

### ELISA Assay for Measuring Acetylated p53 Protein

U2OS cells in six-well plates were pretreated with test compounds for 1 hour followed by doxorubicin treatment at 1 µg/ml for four hours to induce p53 acetylation. For the experiments to evaluate knockdown of SIRT1 on doxorubicin-induced p53 acetylation, SIRT1-siRNA or NT-siRNA was transfected into cells 40 hours prior to compound pretreatment. An ELISA assay was developed and used to measure acetylated p53 protein in cell lysates. After coating an anti-acetylated p53 antibody (Cell Signaling, 2525) onto a 96-well plate at 4°C overnight, the plate was blocked for 2 hours. Cell lysates were then transferred onto the ELISA plate and incubated at RT for 3 hours. Detection of acetylated p53 protein was achieved by using a HRP-labeled anti-p53 antibody (Santa Cruz, sc-126 HRP) followed by exposing the ELISA plate to HRP substrate (BioFX Laboratories, TMBC-0100-01). Absorbance at OD 450 nm was read by a SpectraMax M5 plate reader. The quantity of acetylated p53 protein in samples was calculated by fitting the data into a standard curve of acetylated p53 protein on the same ELISA plate. To compare the levels of acetylated p53 among samples, the quantity of acetylated p53 was normalized against the amount of total protein in the lysates that had been transferred onto the ELISA plate.

### Measurement of Acetylated p65 Protein Induced by TNFα

HEK 293T/17 cells in 10 cm dishes were transfected with p300-HAT plasmid by FuGENE 6 transfection reagent (Roche, 11814443001). 18 hours after transfection, cells were pretreated with compounds for 6 hours and then stimulated with 20 ng/ml recombinant human TNFα for 20 minutes (Invitrogen, PHC3016). Cells were then lysed on plate and the supernatants after centrifugation were subject to immunoprecipitation by an anti-acetylated K310-p65 antibody (Abcam, ab19870) overnight at 4°C with rotating. On the next day, 50 µl Protein A Dynabeads were added to the immunoprecipitation samples and rotated for 3 hours at 4°C. After washing, the immunoprecipitated acetylated p65 protein was extracted by 2X SDS sample buffer and probed for p65 (anti-p65 antibody, Santa Cruz, sc-8008) by western blotting. A phospho-NF-κB (Ser536) antibody (Cell Signaling, 3031) and a phospho-IκBα (Ser32/36) antibody (Cell Signaling, 9246) were used to probe for phosphorylated p65 and phosphorylated IκBα in cell lysates. Densitometry quantitation of acetylated p65 protein on western blots was conducted by using Odyssey software. For the experiments to assess SIRT1 overexpression on TNFα-induced p65 acetylation, pcDNA-hSIRT1 plasmid or empty vector was co-transfected with p300-HAT plasmid at the same time into cells by FuGENE 6 transfection reagent.

### NF-κB Luciferase Reporter Assay

HEK 293 cells stably expressing a luciferase reporter driven by a tandem of 3×κB DNA element were plated onto 384-well plates. On the day of experiment, cells were pretreated with compounds for 1 hour and then stimulated with 50 ng/ml of recombinant TNFα for 3 hours. Luciferase activity was measured by Steady-Glo Luciferase Assay System (Promega, E2550) according to the manufacturer’s protocol. For the experiments to examine SIRT1 overexpression on NF-κB transcriptional activity, SIRT1 plasmid or empty vector was transfected into cells 24 hours prior to TNFα treatment. Cell viability in the sister plate was measured by ATPlite according to the manufacturer’s protocol (PerkinElmer, 6016739).

### Measurement of LPS-induced TNFα Secretion by RAW Cells

RAW 264.7 macrophages were seeded at 4×10^4^ cells per well in 96-well plates. 16 hours after seeding, cells were pretreated with compounds for 1 hour, followed by stimulation with 100 ng/ml LPS (*E. coli*, Calbiochem) for 1 hour. Media was then transferred into TNFα ELISA plates (Invitrogen, KMC3011). TNFα in supernatants was then measured following the manufacturer’s protocol. Cell viability was determined by AlamarBlue according to the manufacturer’s protocol (Invitrogen, DAL1100). For the experiments to evaluate knockdown of SIRT1 on STAC-inhibited TNFα secretion, SIRT1-siRNA or NT-siRNA was transfected into cells 48 hours prior to compound pretreatment.

### Test of SRTCX1003 and Dexamethasone on LPS-induced Cytokine Production *in vivo*

11 weeks old male BALB/c mice purchased from Jackson Laboratory were acclimated with minimum 4 days under the same conditions as for the actual test. Mice weighted at 25–28 grams were randomized into 6 groups with 8 mice each and were orally dosed with vehicle, 10% PEG, 10% VitE-TPGS, or SRTCX1003 at 3 mg/kg, 10 mg/kg, 30 mg/kg, 100 mg/kg, or dexamethasone at 1 mg/kg (Sigma, MO part#D1756, lot 096K1805), all diluted in vehicle of 10% PEG, 10% VitE-TPGS. One hour after oral dose, animals were administered 0.25 mg/kg LPS in PBS (Sigma, MO part# L2630, lot 128K4054) through intravenous injection. Ninety minutes later, animals were sacrificed by CO_2_ asphyxiation, and the blood was collected by cardiac puncture. Plasma was thereafter separated from blood cells by centrifugation for 8 minutes at 13,200 rpm in an Eppendorf using a 5214R centrifuge. A 10-fold dilution of plasma was performed prior to the measurement of TNFα (Invitrogen, CA, part# KMC3011) and IL-12p40 (Invitrogen, CA, part# KMC0121) by ELISA. In parallel, 30 µl of undiluted plasma was submitted for drug exposure analysis by mass spectrometry.

### Ethics Statement

All animal studies were conducted at Sirtris, a GSK Company (Cambridge, MA) following the guidelines of the institutional animal use and care committee (IACUC). All protocols and animal ethics were approved by the Sirtris IACUC. Appropriate measures were taken to minimize distress to the animals, following the guidelines of Sirtris IACUC and GSK institutional animal use policies. The animals were anesthetized using isoflurane prior to intravenous injection of LPS and were allowed to recover for several minutes prior to returning to the cage. Mice were sacrificed by asphyxiation using carbon dioxide followed by cardiac puncture.

## Results

### Development of a Quantitative Assay to Monitor Cellular Acetylated p65 Levels

**Figure 2:**
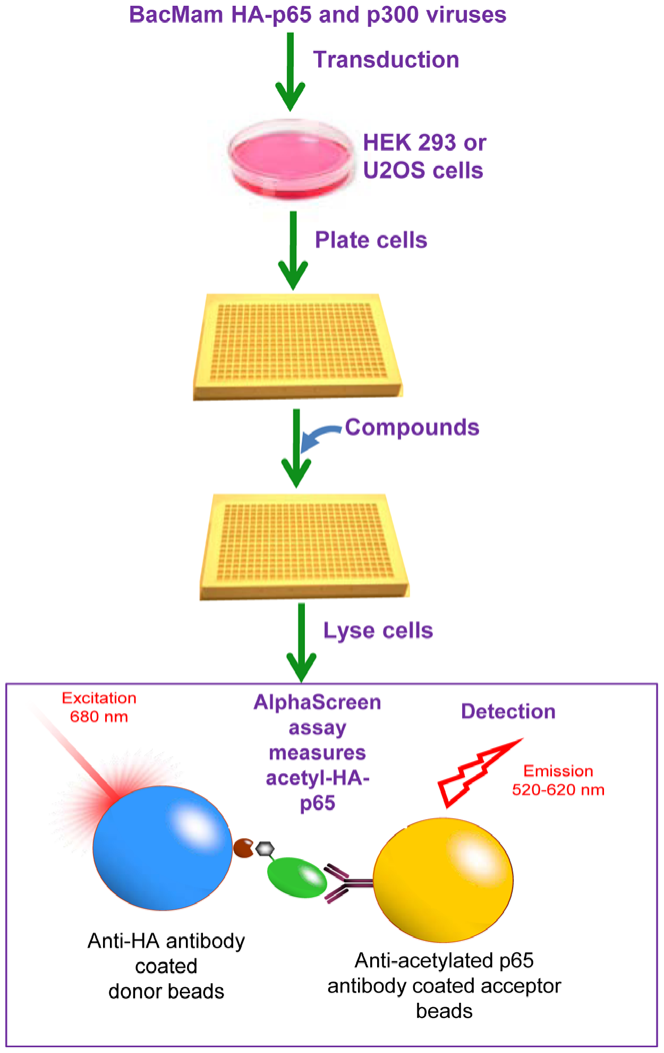
Development of a quantitative assay for measuring cellular acetylated p65 levels. Flow diagram depicts the assay procedure, including BacMam p65 and BacMam p300 viral transduction, plating cells, compound treatment, cell lysis and detection of acetylated p65 protein by AlphaScreen format.

**Figure 3:**
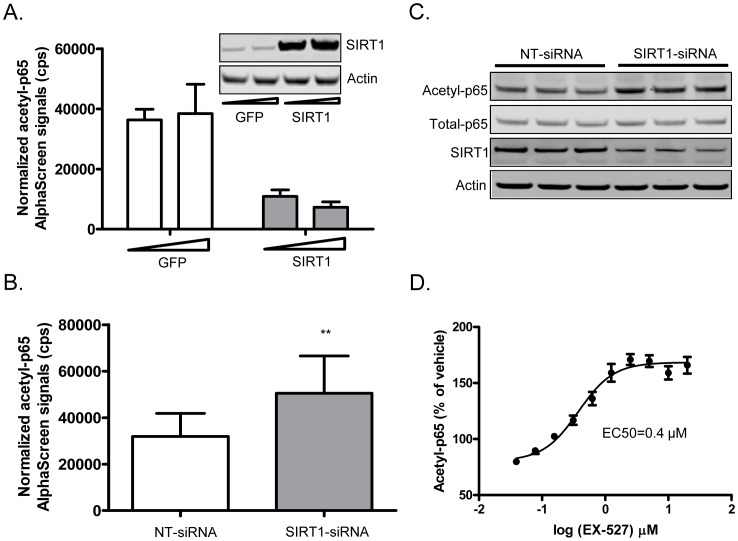
SIRT1 regulates cellular acetylated p65 levels. (A) Levels of acetylated p65 in BacMam SIRT1 virus or BacMam GFP virus transduced cells. Western blot in the right upper corner shows the level of SIRT1 expression upon BacMam SIRT1 virus transduction. (B–C) Levels of acetylated p65 protein in cells transfected with SIRT1-siRNA or NT-siRNA as measured by AlphaScreen assay in (B), or demonstrated by immunoblotting in (C). (D) Dose-response effect of EX-527 on levels of acetylated p65 protein. All error bars represent s.d. of at least 4 replicates. *P<0.05, **P<0.01 and ***P<0.001.

We developed a high throughput cellular assay to examine the ability of compounds to promote SIRT1-mediated deacetylation of p65 protein in cells. This assay system uses BacMam virus transduction in U2OS or HEK 293 cells to co-express p300 HAT and an HA-tagged p65 NF-κB subunit to enable detection of acetylated p65. SIRT1 has been shown to specifically deacetylate p65 at K310 [15], thus we developed the assay to detect acetylated K310-p65 in cell lysates by AlphaScreen format (Figure 2). Using this cellular system, we demonstrated that overexpression of SIRT1 by BacMam virus transduction mediated a reduction of acetylated p65 protein as compared to BacMam GFP virus transduced (control) cells (Figure 3A). Conversely, knockdown of SIRT1 by SIRT1-siRNA reduced SIRT1 by 70% and resulted in elevation of acetylated p65 protein levels compared to samples transfected with non-targeting siRNA (Figure 3B and 3C). Similarly, pharmacological inhibition of SIRT1 by a specific SIRT1 inhibitor, EX-527 [46], increased the levels of acetylated p65 protein (Figure 3D). These data indicate that SIRT1 regulates p65 acetylation in cells and this cellular assay is suitable for profiling STACs.

### SIRT1-activating Compounds (STACs) Activate SIRT1 Deacetylase Activity *in vitro*

**Figure 4:**
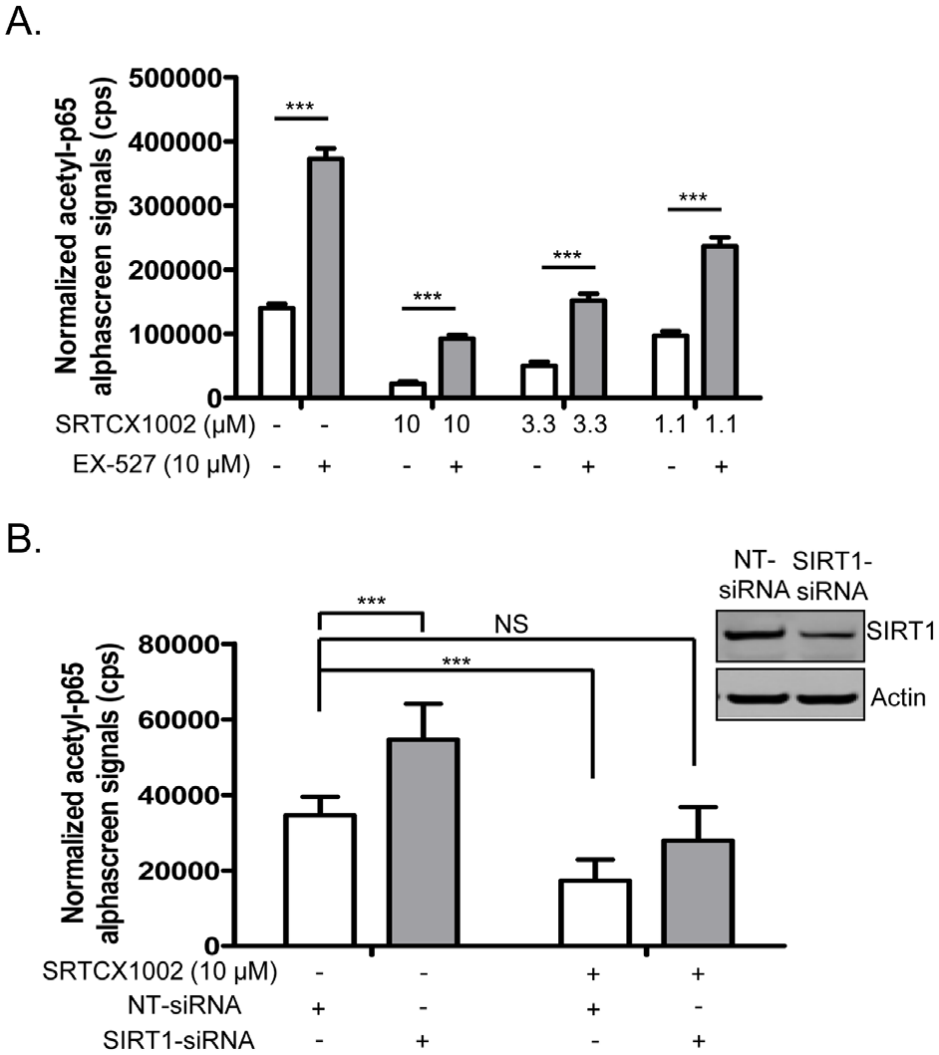
STACs promote SIRT1-mediated deacetylation of p65 protein in cells. (A) Levels of acetylated p65 in U2OS cells treated with varied concentrations of SRTCX1002 with or without 10 µM EX-527. (B) Levels of acetylated p65 in vehicle or 10 µM SRTCX1002 treated U2OS cells transfected with either SIRT1-siRNA or NT-siRNA. Western blots in the right upper corner indicate that SIRT1 was knocked down by 70% by SIRT1-siRNA. All error bars represent s.d. of at least 4 replicates. *P<0.05, **P<0.01 and ***P<0.001.

STACs that were identified from biochemical assays (File S1) were evaluated in the acetylated p65 cellular assay. Two representatives from the benzimidazole compound series (SRTCX1002 and SRTCX1003) shown in Figure 1A and two representatives from the quinolone compound series (SRTCD1023, SRTCL1015) (core structure shown in Figure 1B) and a structurally related inactive control compound that does not activate SIRT1 in the biochemical assays for each series (SRTCZ1001 and SRTCE1022, respectively), were tested in the cellular p65 acetylation assay for their effects on regulating p65 acetylation. Data showed that all of the four STACs that biochemically activated SIRT1 *in vitro* could mediate a dose-dependent reduction of acetylated p65 protein (Figure 4A, S1 and Table S1) with the two biochemically inactive compounds showing no effect (Table S1). The compounds were tested for the inhibition of histone acetyltransferases (HATs) since this could result in a reduction of acetylated p65 that could not be distinguished from an enhancement of deacetylation. SRTCX1002 and SRTCX1003 showed poor inhibition of CBP and p300 with IC_50_s of 24 µM and 11 µM respectively (data not shown), potencies which are significantly lower than their IC_50_s for reducing levels of acetylated p65 in the cellular assay (Table S1). Furthermore, the two compounds from the other chemical series, SRTCD1023 and SRTCL1015, did not inhibit any of the HAT proteins up to the maximum concentration used (30 µM, data not shown). This indicates that STAC-mediated reduction of acetylated p65 cannot be attributed to HAT inhibition.

To see whether the reduction of acetylated p65 protein by STACs is mediated through SIRT1, levels of acetylated p65 protein in vehicle or STAC-treated cells with or without 10 µM EX-527 were measured. Our data showed that inhibition of SIRT1 by EX-527 significantly reversed STAC-mediated reduction of acetylated p65 (Figure 4A, Figure S1A−C), indicating that the STAC effect is SIRT1-dependent. This conclusion was reinforced following the knockdown of SIRT1 expression by siRNA that resulted in a reversal of compound mediated reduction of acetylated p65 (Figure 4B). Since p53 is a well-established SIRT1 substrate [48], [49], we also tested the effect of STACs on acetylated p53 levels in cells treated with doxorubicin. Our data showed that treatment of SRTCX1002 dose-dependently reduced doxorubicin-induced p53 acetylation, and this reduction was largely reversed upon inhibition of SIRT1 by EX-527 or knockdown of SIRT1 expression by siRNA (Figure S2). SRTCX1003 was also tested and showed a similar effect (data not shown). Taken together, our data indicate that STAC-mediated reduction of acetylated p65 or p53 protein is dependent on SIRT1.

### STACs Attenuate TNFα-induced p65 Acetylation in Cells

**Figure 5:**
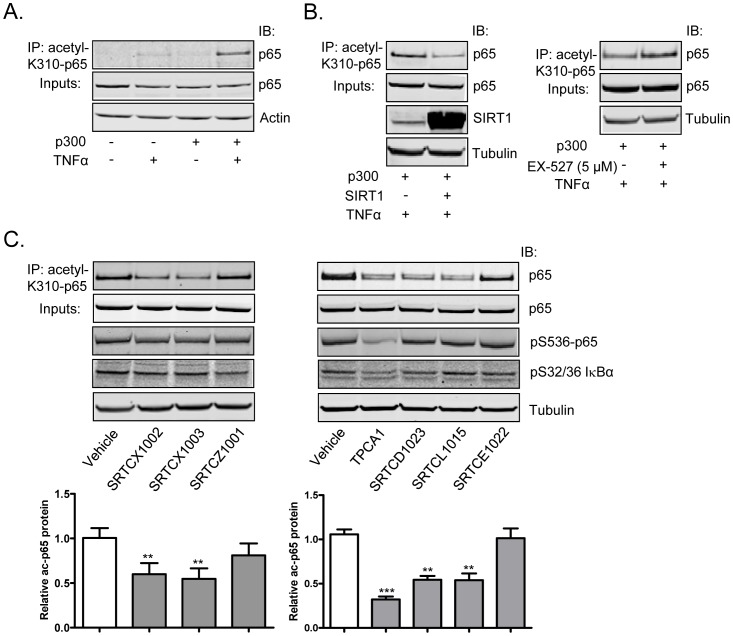
STACs attenuate TNFα-induced p65 acetylation. (A) Western blots of immunoprecipitated acetylated K310-p65 protein, p65 and actin protein in whole cell lysates from vector or p300 transfected cells with or without TNFα stimulation. (B) Left panel, western blots of immunoprecipitated acetylated K310-p65 protein, SIRT1, p65 and tubulin in whole cell lysates from TNFα-stimulated cells transfected with p300 plus empty vector or p300 plus SIRT1. Right panel, western blots of immunoprecipitated acetylated K310-p65 protein, p65 and tubulin in whole cell lysates from TNFα-stimulated p300 overexpressing cells that were pretreated with EX-527 for 6 hours. (C) Upper panels, western blots of immunoprecipitated acetylated K310-p65 protein, p65, phospho-NF-κB (Ser536), phospho-IκBα (Ser32/Ser36) and tubulin in whole cell lysates from cells pretreated with compounds for 6 hours followed by 20 minutes TNFα stimulation. Lower panels, densitometry quantitation of the western blot for immunoprecipitated acetylated p65 protein. The level of acetylated p65 in TNFα-stimulated and vehicle treated sample was set as 1. Experiments were repeated at least 2 times and western blots from one experiment were shown as representative. Error bars present s.d. of densitometry quantitation of western blots from at least two experiments. *P<0.05, **P<0.01 and ***P<0.001.

We wanted to investigate the effect of STACs on a physiological proinflammatory stimulus since the assay described above relies on the overexpression of p65 and p300. It has been shown that TNFα stimulation can induce p65 acetylation, which can in turn be reduced by SIRT1 deacetylase activity [22], [50]. Indeed, we found that treating HEK 293T/17 cells with TNFα induced a very modest level of p65 acetylation at K310. These levels of acetylated p65 were significantly enhanced when p300 protein was expressed in these cells (Figure 5A). p300 overexpression in the absence of TNFα stimulation did not induce any detectable levels of acetylated p65, as TNFα is required for IκBα degradation and p65 translocation to the nucleus to be acetylated by p300. Because TNFα stimulation combined with p300 overexpression induces a readily-detectable level of acetylated p65, we studied the effects of SIRT1 overexpression or activation by STACs on this system for the following experiments. Consistent with previous reports, overexpression of SIRT1 attenuated (Figure 5B), whereas inhibition of SIRT1 activity by EX-527 increased p65 acetylation stimulated by TNFα (Figure 5B), indicating that SIRT1 is a primary p65 deacetylase in response to TNFα stimulation.

Next we tested whether STACs could affect TNFα-induced p65 acetylation. We pretreated HEK 293T/17 cells with each of the STACs for 6 hours and then stimulated them with TNFα. The western blot of the immunoprecipitated acetylated p65 protein from these samples showed that STACs significantly reduced TNFα-stimulated p65 acetylation, while the two biochemically inactive compounds showed no effect (Figure 5C). A specific IKK2 inhibitor, TPCA1 [51], was also included as a positive control. As expected, TPCA1 significantly inhibited TNFα-induced p65 acetylation by preventing p65 nuclear translocation and its subsequent acetylation (Figure 5C). Besides acetylation, NF-κB activation is regulated by phosphorylation, and interplay exists between phosphorylation and acetylation of NF-κB. For example, phosphorylation of Ser536 has been shown to enhance p65 acetylation [52]. To see whether STACs could affect IKK-IκB signaling pathways regulating p65 phosphorylation upstream of p65 acetylation, we probed STAC-treated samples with antibodies recognizing phospho-NF-κB (Ser536) and phospho-IκBα (Ser32/Ser36). As shown in Figure 5C, while TPCA1 diminished TNFα-stimulated phosphorylation of p65 at serine 536 and phosphorylation of IκBα at serine 32 and serine 34, STACs did not have any effect on phosphorylation of these residues. These data suggest that STAC-mediated reduction of acetylated p65 protein occurs downstream of IKK and IκB.

### STACs Suppress TNFα-induced NF-κB Transcriptional Activation

**Figure 6:**
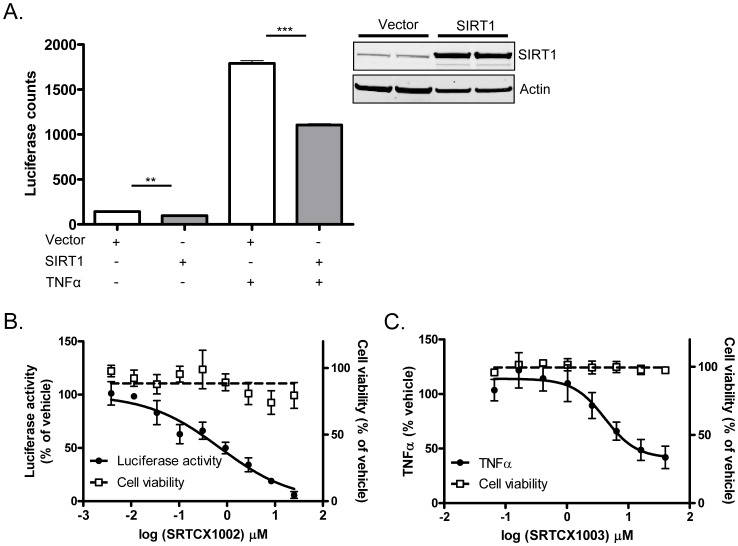
STACs suppress stimuli-induced NF-κB transcriptional activation and cytokine secretion. (A) NF-κB luciferase reporter counts in HEK 293 cells transfected with SIRT1 or empty vector with or without TNFα stimulation. Western blot in the right upper corner indicated SIRT1 expression induced by SIRT1 plasmid transfection. (B) Dose-response effect of SRTCX1002 on NF-κB luciferase reporter activity in HEK293 cells stimulated with TNFα. Error bars represent s.d. of at least four replicates. (C) Dose-response effect of SRTCX1003 on LPS-induced TNFα secretion from RAW cells. *P<0.05, **P<0.01 and ***P<0.001.

It has been shown previously that acetylation of p65 at lysine 310 is required for full activation of NF-κB function [7]. To investigate whether the reduction of p65 acetylation induced by SIRT1 overexpression or SIRT1 activation by STACs could lead to suppression of NF-κB transcriptional activation, we measured NF-κB luciferase reporter activity in HEK 293 cells overexpressing SIRT1 or pretreated with STACs prior to TNFα stimulation. Our data showed that overexpression of SIRT1 suppressed basal and TNFα-stimulated NF-κB transcriptional activity as measured by an NF-κB luciferase reporter gene assay (Figure 6A). Likewise, treatment of STACs induced a dose-dependent reduction of TNFα-stimulated NF-κB transcriptional activation while the inactive compounds had no effect (Figure 6B and Table 1), consistent with the effect exerted by SIRT1 overexpression (Figure 6A).

**Table 1:** STACs suppress stimuli-induced NF-κB transcriptional activation and cytokine secretion.

Compound ID	NF-êB luciferase reporter assay IC_50_ (µM)	LPS-induced TNFá secretion assay IC_50_ (µM)
SRTCX1002	0.71	7.58
SRTCX1003	0.95	12.52
SRTCZ1001	>20	>40
SRTCD1023	3.30	3.16
SRTCL1015	1.24	3.41
SRTCE1022	>20	>40

Table shows the IC_50_ values of the STACs in NF-κB luciferase reporter assay and LPS-induced TNFα secretion assay.

### STACs Reduce LPS-induced Cytokine Secretion from RAW Cells

As NF-κB plays a significant role in regulating inflammatory cytokine production, we next determined whether STACs could block LPS-induced TNFα secretion from RAW 264.7 murine macrophage cells. As shown in Figure 6C and Table 1, all of the four STACs showed dose-dependent reduction of LPS-induced TNFα secretion from RAW cells, whereas the control compounds showed no effect. Further, the reduction of TNFα secretion from RAW cells mediated by SRTCX1003 treatment was largely reversed upon knockdown of SIRT1 by siRNA (Figure S3), indicating that STAC-mediated inhibition of LPS-induced inflammatory response is dependent on SIRT1.

### SRTCX1003 Decreases LPS-stimulated Cytokine Production *in vivo*

**Figure 7:**
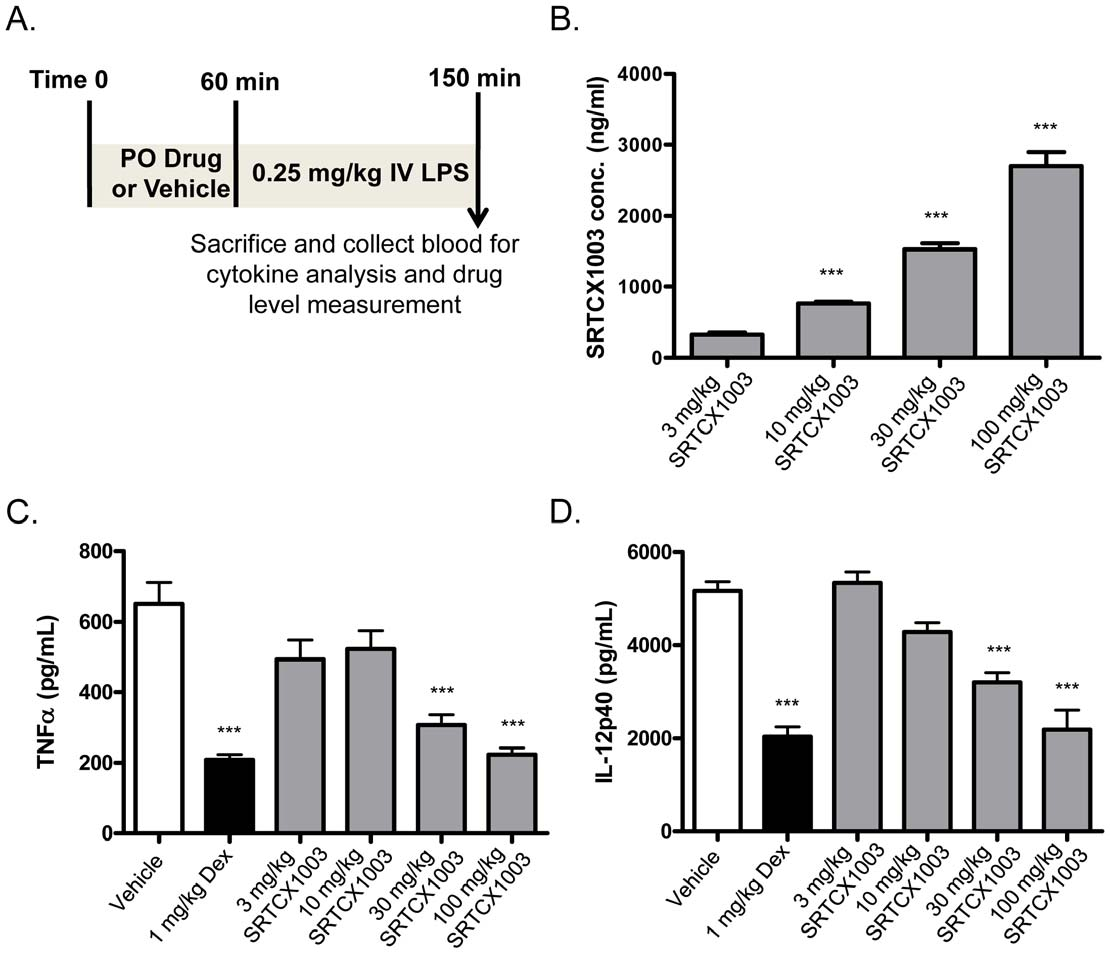
SRTCX1003 decreases LPS-stimulated cytokine production *in vivo*. (A) Schematic illustration of the acute LPS-induced inflammation mouse model. (B) Compound plasma concentration of mice dosed with SRTCX1003 for 2.5 hours. (C–D) Dose-response effect of SRTCX1003 and the effect of 1 mg/kg dexamethasone on LPS-induced production of TNFα in (C) and IL-12p40 in (D). Error bars represent s.d. of eight mice in each group. Please note that reduction of TNFα and IL-12p40 by SRTCX1003 at 100 mg/kg was comparable to that induced by 1 mg/kg dexamethasone. *P<0.05, **P<0.01 and ***P<0.001.

Previous studies on SRT1720, SRT2530 and SRT2379 have demonstrated that these earlier generation STACs can improve insulin sensitivity in mice fed with high fat diet in a SIRT1-dependent manner, which is at least partially due to their anti-inflammatory effects [37], [38]. To examine the effects of STACs on acute systemic inflammation, we used a LPS-induced inflammation mouse model as depicted in Figure 7A. As reported previously [53], [54], plasma concentration of a number of inflammatory cytokines such as TNFα and IL-12 was rapidly elevated following intravenous LPS administration. TNFα, in particular, increased by >1000 fold, 90 minutes after the LPS administration, in agreement with previous reports [53]. We hypothesized that pretreatment of the animals with an orally bioavailable STAC should blunt the LPS-induced inflammatory cytokines. An oral dose of 3, 10, 30 or 100 mg/kg SRTCX1003 or 1 mg/kg of the positive control dexamethasone was administered to the mice 60 minutes prior to LPS administration. Plasma concentration of SRTCX1003 at the time of sacrificing was measured which showed a dose-response drug exposure of SRTCX1003 in mice (Figure 7B). Consistently, SRTCX1003 treatment dose-dependently reduced LPS-stimulated TNFα and IL-12p40 production. Strikingly, SRTCX1003 at the highest dose of 100 mg/kg showed an efficacy that was comparable to dexamethasone (Figure 7C and 7D).

## Discussion

Inflammation has been shown to play a major role in contributing to the pathology of many chronic diseases, including COPD, rheumatoid arthritis, T2D, cancer, Alzheimer’s disease, cardiovascular, and renal diseases among others [55]. Use of steroids and nonsteroidal anti-inflammatory drugs (NSAIDs) is not always appropriate or optimal for the chronic treatment of these age-related diseases due to their undesired side effects [56], [57]. Furthermore, the current anti-inflammatory drugs do not help to restore the dysregulated physiological functions in diseases such as diabetes through direct modulation of dysregulated pathways. Therefore, there is a clear and in some cases unmet medical need for developing more effective and mechanistically-targeted anti-inflammatory drugs.

The NAD^+^-dependent deacetylase SIRT1 has been shown to regulate the inflammatory response to multiple stimuli, as well as improve metabolic, neuronal, cardiovascular and renal functions in diseases of aging, thus validating this deacetylase as a potential therapeutic target for drug discovery and development [58], [59]. We have shown here that representatives of two distinct chemical classes of STACs activate SIRT1 *in vitro* and promote SIRT1-mediated deacetylation of p65 protein in cells leading to the suppression of NF-κB transcriptional activation stimulated by the proinflammatory cytokine TNFα. Moreover, treatment of inflammatory cells with the STACs mediates a reduction of LPS-induced TNFα secretion while oral administration to mice challenged with LPS suppresses this cytokine and IL-12 *in vivo*.

Mutational analyses of p65 have revealed the importance of lysine 310 acetylation on NF-κB activation [7], [50]. The AlphaScreen format assay described herein allows quantitative assessment of the effect of small molecules on SIRT1-dependent deacetylation of K310 on p65. While this system relies on the overexpression of both p300 HAT and p65 for optimal signal to noise measurements, we have demonstrated that the effect of compounds in this assay is relevant, as the same impact can be observed on endogenous p65 in cells stimulated with TNFα. Further, STAC treatment of cells attenuates TNFα-induced p65 acetylation without affecting IKK-mediated phosphorylation of p65 and IκBα, indicating that STAC-mediated p65 deacetylation is downstream of IKK activation.

It is possible that other sirtuins could affect p65 acetylation. SIRT2 has also been shown to deacetylate p65 and regulate NF-κB [60]. We have tested the STACs, and find that they do not activate SIRT2 (data not shown). In other studies, SIRT6 has been shown to affect NF-κB-dependent gene expression, but through an indirect means of deacetylating histone H3, which affects recruitment of NF-κB [61]. It is also not completely clear whether SIRT6 activity would regulate NF-κB, given one study which showed SIRT6 suppressed NF-κB targeted gene expression [61] while another study did not detect changes in NF-κB regulated gene expression in muscles of SIRT6 deficient mice [62]. Also under investigation is the effect of STACs on AP-1 and/or T cell function, which are also potentially regulated by SIRT1 and may contribute to the suppression of inflammatory response [63], [64].

Previous studies have already demonstrated the effects of STACs on regulating the inflammation in liver and adipose that is associated with high fat diet and obesity [37], [38], [41]. The studies reported here describe the effect of STACs on an acute systemic inflammatory response. SRTCX1003 shows dose-dependent reduction of TNFα and IL-12 production in the plasma of mice treated with LPS. Strikingly, SRTCX1003 treatment at the dose of 100 mg/kg showed an efficacy that is comparable to dexamethasone. We have tested and found that SRTCX1003 has no direct activity on the glucocorticoid receptor. It will be of interest to evaluate the therapeutic potential of combining SRTCX1003 with dexamethasone, as well as other anti-inflammatory modulators on combating inflammation. Experimental medicine studies of a STAC that has been evaluated for its *in vivo* profile shows results in humans consistent with the observations in rodents. That is, human subjects that were dosed with STAC SRT2104, then subsequently challenged with a low dose of LPS, have significantly suppressed levels of inflammatory cytokines IL-6 and IL-8, and attenuated levels of TNFα, compared to the subjects on placebo [65].

The efficacy seen in the LPS model prompts the question of whether STACs would show therapeutic benefit in inflammatory disease models. Preliminary studies of STACs on disease models, such as dextran sulfate sodium (DSS)-induced inflammatory bowel disease (IBD) show that STAC treatment reduces colonic inflammation in the IBD model (JL Ellis, unpublished data). Safety assessment studies on STACs are ongoing to assess whether STACs can be tested in chronic inflammation disease models, and to support progression of STACs into clinical trials. Collectively, these findings underscore the promise of SIRT1 modulators as a novel therapeutic approach for inflammatory diseases.

## Supporting Information

Figure S1 STACs promote SIRT1-mediated deacetylation of p65 protein. (A–C) Treatment of SRTCX1003, SRTCD1023 or SRTCL1015 at varied doses with or without 10 µM EX-527 on levels of acetylated p65 protein in U2OS cells. All error bars represent s.d. of at least 4 replicates. *P<0.05, **P<0.01 and ***P<0.001.
(TIF)

Figure S2 SRTCX1002 enhances SIRT1-mediated deacetylation of p53 protein. **(**A) Effects of varied concentrations of SRTCX1002 with or without 10 µM EX-527 on doxorubicin-induced p53 acetylation in U2OS cells. (B) Effects of SRTCX1002 on doxorubicin-induced p53 acetylation in U2OS cells transfected with either SIRT1-siRNA or NT-siRNA. Western blots in the right upper corner indicate that SIRT1 was knocked down by 70% by SIRT1-siRNA. All error bars represent s.d. of at least 4 replicates. *P<0.05, **P<0.01 and ***P<0.001.
(TIF)

Figure S3 SRTCX1003 reduces LPS-induced TNFα secretion from RAW cells via SIRT1. Effects of SRTCX1003 on LPS-induced TNFα secretion from RAW cells transfected with either SIRT1-siRNA or NT-siRNA. Western blots in the right upper corner indicate that SIRT1 was knocked down by 70% by SIRT1-siRNA. All error bars represent s.d. of at least 3 replicates. *P<0.05, **P<0.01 and ***P<0.001.
(TIF)

File S1 Materials and Methods.
(DOCX)

Table S1 STACs activate SIRT1 deacetylase activity *in vitro*. Table shows the EC1.5 and Max Act values of the STACs in SIRT1 enzymatic assay with p53-TAMRA peptide substrate and IC_50_ values of the STACs in the cellular p65 assay.
(DOCX)

## References

[1] Chung HY, Lee EK, Choi YJ, Kim JM, Kim DH, et al (2011) Molecular inflammation as an underlying mechanism of the aging process and age-related diseases. J Dent Res 90: 830–840. 21447699 10.1177/0022034510387794

[2] Goh FG, Midwood KS (2012) Intrinsic danger: activation of Toll-like receptors in rheumatoid arthritis. Rheumatology (Oxford) 51: 7–23. 21984766 10.1093/rheumatology/ker257

[3] Viatour P, Merville MP, Bours V, Chariot A (2005) Phosphorylation of NF-kappaB and IkappaB proteins: implications in cancer and inflammation. Trends Biochem Sci 30: 43–52. 15653325 10.1016/j.tibs.2004.11.009

[4] Ishinaga H, Jono H, Lim JH, Kweon SM, Xu H, et al (2007) TGF-beta induces p65 acetylation to enhance bacteria-induced NF-kappaB activation. EMBO J 26: 1150–1162. 17268554 10.1038/sj.emboj.7601546 PMC1852843

[5] Yang XD, Huang B, Li M, Lamb A, Kelleher NL, et al (2009) Negative regulation of NF-kappaB action by Set9-mediated lysine methylation of the RelA subunit. EMBO J 28: 1055–1066. 19262565 10.1038/emboj.2009.55 PMC2683704

[6] Ghizzoni M, Haisma HJ, Maarsingh H, Dekker FJ (2011) Histone acetyltransferases are crucial regulators in NF-kappaB mediated inflammation. Drug Discov Today 16: 504–511. 21477662 10.1016/j.drudis.2011.03.009 PMC5218544

[7] Chen LF, Mu Y, Greene WC (2002) Acetylation of RelA at discrete sites regulates distinct nuclear functions of NF-kappaB. EMBO J 21: 6539–6548. 12456660 10.1093/emboj/cdf660 PMC136963

[8] Kiernan R, Bres V, Ng RW, Coudart MP, El Messaoudi S, et al (2003) Post-activation turn-off of NF-kappa B-dependent transcription is regulated by acetylation of p65. J Biol Chem 278: 2758–2766. 12419806 10.1074/jbc.M209572200

[9] Rothgiesser KM, Fey M, Hottiger MO (2010) Acetylation of p65 at lysine 314 is important for late NF-kappaB-dependent gene expression. BMC Genomics 11: 22. 20064247 10.1186/1471-2164-11-22 PMC2823688

[10] Buerki C, Rothgiesser KM, Valovka T, Owen HR, Rehrauer H, et al (2008) Functional relevance of novel p300-mediated lysine 314 and 315 acetylation of RelA/p65. Nucleic Acids Res 36: 1665–1680. 18263619 10.1093/nar/gkn003 PMC2275151

[11] Chen L, Fischle W, Verdin E, Greene WC (2001) Duration of nuclear NF-kappaB action regulated by reversible acetylation. Science 293: 1653–1657. 11533489 10.1126/science.1062374

[12] Sheppard KA, Phelps KM, Williams AJ, Thanos D, Glass CK, et al (1998) Nuclear integration of glucocorticoid receptor and nuclear factor-kappaB signaling by CREB-binding protein and steroid receptor coactivator-1. J Biol Chem 273: 29291–29294. 9792627 10.1074/jbc.273.45.29291

[13] Sheppard KA, Rose DW, Haque ZK, Kurokawa R, McInerney E, et al (1999) Transcriptional activation by NF-kappaB requires multiple coactivators. Mol Cell Biol 19: 6367–6378. 10454583 10.1128/mcb.19.9.6367 PMC84607

[14] Zhong H, May MJ, Jimi E, Ghosh S (2002) The phosphorylation status of nuclear NF-kappa B determines its association with CBP/p300 or HDAC-1. Mol Cell 9: 625–636. 11931769 10.1016/s1097-2765(02)00477-x

[15] Yeung F, Hoberg JE, Ramsey CS, Keller MD, Jones DR, et al (2004) Modulation of NF-kappaB-dependent transcription and cell survival by the SIRT1 deacetylase. Embo J 23: 2369–2380. 15152190 10.1038/sj.emboj.7600244 PMC423286

[16] Haigis MC, Sinclair DA (2010) Mammalian sirtuins: biological insights and disease relevance. Annu Rev Pathol 5: 253–295. 20078221 10.1146/annurev.pathol.4.110807.092250 PMC2866163

[17] Baur JA (2010) Resveratrol, sirtuins, and the promise of a DR mimetic. Mech Ageing Dev 131: 261–269. 20219519 10.1016/j.mad.2010.02.007 PMC2862768

[18] Chen J, Zhou Y, Mueller-Steiner S, Chen LF, Kwon H, et al.. (2005) SIRT1 protects against microglia-dependent beta amyloid toxicity through inhibiting NF-kappa B signaling. J Biol Chem. 10.1074/jbc.M509329200 16183991

[19] Yang SR, Wright J, Bauter M, Seweryniak K, Kode A, et al (2007) Sirtuin regulates cigarette smoke-induced proinflammatory mediator release via RelA/p65 NF-kappaB in macrophages in vitro and in rat lungs in vivo: implications for chronic inflammation and aging. Am J Physiol Lung Cell Mol Physiol 292: L567–576. 17041012 10.1152/ajplung.00308.2006

[20] Rajendrasozhan S, Yang SR, Kinnula VL, Rahman I (2008) SIRT1, an antiinflammatory and antiaging protein, is decreased in lungs of patients with chronic obstructive pulmonary disease. Am J Respir Crit Care Med 177: 861–870. 18174544 10.1164/rccm.200708-1269OC PMC2292827

[21] Pfluger PT, Herranz D, Velasco-Miguel S, Serrano M, Tschop MH (2008) Sirt1 protects against high-fat diet-induced metabolic damage. Proc Natl Acad Sci U S A 105: 9793–9798. 18599449 10.1073/pnas.0802917105 PMC2474520

[22] Schug TT, Xu Q, Gao H, Peres-da-Silva A, Draper DW, et al (2010) Myeloid deletion of SIRT1 induces inflammatory signaling in response to environmental stress. Mol Cell Biol 30: 4712–4721. 20647536 10.1128/MCB.00657-10 PMC2950528

[23] Kim EJ, Kho JH, Kang MR, Um SJ (2007) Active regulator of SIRT1 cooperates with SIRT1 and facilitates suppression of p53 activity. Mol Cell 28: 277–290. 17964266 10.1016/j.molcel.2007.08.030

[24] Kim JE, Chen J, Lou Z (2008) DBC1 is a negative regulator of SIRT1. Nature 451: 583–586. 18235501 10.1038/nature06500

[25] Zhao W, Kruse JP, Tang Y, Jung SY, Qin J, et al (2008) Negative regulation of the deacetylase SIRT1 by DBC1. Nature 451: 587–590. 18235502 10.1038/nature06515 PMC2866287

[26] Kwon HS, Brent MM, Getachew R, Jayakumar P, Chen LF, et al (2008) Human immunodeficiency virus type 1 Tat protein inhibits the SIRT1 deacetylase and induces T cell hyperactivation. Cell Host Microbe 3: 158–167. 18329615 10.1016/j.chom.2008.02.002 PMC2680745

[27] Revollo JR, Grimm AA, Imai S (2004) The NAD biosynthesis pathway mediated by nicotinamide phosphoribosyltransferase regulates Sir2 activity in mammalian cells. J Biol Chem 279: 50754–50763. 15381699 10.1074/jbc.M408388200

[28] Kang H, Jung JW, Kim MK, Chung JH (2009) CK2 is the regulator of SIRT1 substrate-binding affinity, deacetylase activity and cellular response to DNA-damage. PLoS One 4: e6611. 19680552 10.1371/journal.pone.0006611 PMC2721681

[29] Guo X, Williams JG, Schug TT, Li X (2010) DYRK1A and DYRK3 promote cell survival through phosphorylation and activation of SIRT1. J Biol Chem 285: 13223–13232. 20167603 10.1074/jbc.M110.102574 PMC2857074

[30] Gerhart-Hines Z, Dominy JE Jr, Blattler SM, Jedrychowski MP, Banks AS, et al (2011) The cAMP/PKA Pathway Rapidly Activates SIRT1 to Promote Fatty Acid Oxidation Independently of Changes in NAD(+). Mol Cell 44: 851–863. 22195961 10.1016/j.molcel.2011.12.005 PMC3331675

[31] Escande C, Chini CC, Nin V, Dykhouse KM, Novak CM, et al (2010) Deleted in breast cancer-1 regulates SIRT1 activity and contributes to high-fat diet-induced liver steatosis in mice. J Clin Invest 120: 545–558. 20071779 10.1172/JCI39319 PMC2810074

[32] Yoshino J, Mills KF, Yoon MJ, Imai S (2011) Nicotinamide mononucleotide, a key NAD(+) intermediate, treats the pathophysiology of diet- and age-induced diabetes in mice. Cell Metab 14: 528–536. 21982712 10.1016/j.cmet.2011.08.014 PMC3204926

[33] Milne JC, Lambert PD, Schenk S, Carney DP, Smith JJ, et al (2007) Small molecule activators of SIRT1 as therapeutics for the treatment of type 2 diabetes. Nature 450: 712–716. 18046409 10.1038/nature06261 PMC2753457

[34] Howitz KT, Bitterman KJ, Cohen HY, Lamming DW, Lavu S, et al (2003) Small molecule activators of sirtuins extend Saccharomyces cerevisiae lifespan. Nature 425: 191–196. 12939617 10.1038/nature01960

[35] Baur JA, Sinclair DA (2006) Therapeutic potential of resveratrol: the in vivo evidence. Nat Rev Drug Discov 5: 493–506. 16732220 10.1038/nrd2060

[36] Price NL, Gomes AP, Ling AJ, Duarte FV, Martin-Montalvo A, et al (2012) SIRT1 is required for AMPK activation and the beneficial effects of resveratrol on mitochondrial function. Cell Metab 15: 675–690. 22560220 10.1016/j.cmet.2012.04.003 PMC3545644

[37] Yoshizaki T, Milne JC, Imamura T, Schenk S, Sonoda N, et al (2009) SIRT1 exerts anti-inflammatory effects and improves insulin sensitivity in adipocytes. Mol Cell Biol 29: 1363–1374. 19103747 10.1128/MCB.00705-08 PMC2643824

[38] Yoshizaki T, Schenk S, Imamura T, Babendure JL, Sonoda N, et al (2010) SIRT1 inhibits inflammatory pathways in macrophages and modulates insulin sensitivity. Am J Physiol Endocrinol Metab 298: E419–428. 19996381 10.1152/ajpendo.00417.2009 PMC2838524

[39] Smith JJ, Kenney RD, Gagne DJ, Frushour BP, Ladd W, et al (2009) Small molecule activators of SIRT1 replicate signaling pathways triggered by calorie restriction in vivo. BMC Syst Biol 3: 31. 19284563 10.1186/1752-0509-3-31 PMC2660283

[40] Yamazaki Y, Usui I, Kanatani Y, Matsuya Y, Tsuneyama K, et al.. (2009) Treatment with SRT1720, a SIRT1 Activator, Ameliorates Fatty Liver with Reduced Expression of Lipogenic Enzymes in MSG Mice. Am J Physiol Endocrinol Metab. 10.1152/ajpendo.90997.2008 19724016

[41] Minor RK, Baur JA, Gomes AP, Ward TM, Csiszar A, et al.. (2011) SRT1720 improves survival and healthspan of obese mice. Sci Rep 1. 10.1038/srep00070 PMC3216557 22355589

[42] Vu C, Disch JS, Ng PY, Blum CA, Perni RB (2010) Benzimidazoles and Related Analogs as Sirtuin Modulators. World Intellectual Property Organization Patent WO 2010/003048.

[43] Disch JS, Vu CB, McPherson L, Ng PY, Bemis JE, et al.. (2010) 2-Aryl-Benzimidazole-4-carboxamides as Novel SIRT1 Activators. 240th ACS National Meeting MEDI: 70.

[44] Vu C, Oalmann C, White B, Perni RB (2010) Quinazolinone, Quinolone and Related Analogs as Sirtuin Modulators. World Intellectual Property Organization Patent WO 2010/037129.

[45] Lysko PG, Weinstock J, Webb CL, Brawner ME, Elshourbagy NA (1999) Identification of a small-molecule, nonpeptide macrophage scavenger receptor antagonist. J Pharmacol Exp Ther 289: 1277–1285. 10336517

[46] Solomon JM, Pasupuleti R, Xu L, McDonagh T, Curtis R, et al (2006) Inhibition of SIRT1 catalytic activity increases p53 acetylation but does not alter cell survival following DNA damage. Mol Cell Biol 26: 28–38. 16354677 10.1128/MCB.26.1.28-38.2006 PMC1317617

[47] Robers MB, Loh C, Carlson CB, Yang H, Frey EA, et al (2011) Measurement of the cellular deacetylase activity of SIRT1 on p53 via LanthaScreen(R) technology. Mol Biosyst 7: 59–66. 20931131 10.1039/c0mb00026d

[48] Luo J, Nikolaev AY, Imai S, Chen D, Su F, et al (2001) Negative control of p53 by Sir2alpha promotes cell survival under stress. Cell 107: 137–148. 11672522 10.1016/s0092-8674(01)00524-4

[49] Vaziri H, Dessain SK, Ng Eaton E, Imai SI, Frye RA, et al (2001) hSIR2(SIRT1) functions as an NAD-dependent p53 deacetylase. Cell 107: 149–159. 11672523 10.1016/s0092-8674(01)00527-x

[50] Yang XD, Tajkhorshid E, Chen LF (2010) Functional interplay between acetylation and methylation of the RelA subunit of NF-kappaB. Mol Cell Biol 30: 2170–2180. 20160011 10.1128/MCB.01343-09 PMC2863596

[51] Podolin PL, Callahan JF, Bolognese BJ, Li YH, Carlson K, et al (2005) Attenuation of murine collagen-induced arthritis by a novel, potent, selective small molecule inhibitor of IkappaB Kinase 2, TPCA-1 (2-[(aminocarbonyl)amino]-5-(4-fluorophenyl)-3-thiophenecarboxamide), occurs via reduction of proinflammatory cytokines and antigen-induced T cell Proliferation. J Pharmacol Exp Ther 312: 373–381. 15316093 10.1124/jpet.104.074484

[52] Chen LF, Williams SA, Mu Y, Nakano H, Duerr JM, et al (2005) NF-kappaB RelA phosphorylation regulates RelA acetylation. Mol Cell Biol 25: 7966–7975. 16135789 10.1128/MCB.25.18.7966-7975.2005 PMC1234328

[53] Yang B, Trump RP, Shen Y, McNulty JA, Clifton LG, et al (2008) RU486 did not exacerbate cytokine release in mice challenged with LPS nor in db/db mice. BMC Pharmacol 8: 7. 18474108 10.1186/1471-2210-8-7 PMC2396158

[54] van der Poll T, Jansen PM, Montegut WJ, Braxton CC, Calvano SE, et al (1997) Effects of IL-10 on systemic inflammatory responses during sublethal primate endotoxemia. J Immunol 158: 1971–1975. 9029140

[55] Manabe I (2011) Chronic inflammation links cardiovascular, metabolic and renal diseases. Circ J 75: 2739–2748. 22067929 10.1253/circj.cj-11-1184

[56] Aulakh R, Singh S (2008) Strategies for minimizing corticosteroid toxicity: a review. Indian J Pediatr 75: 1067–1073. 19023531 10.1007/s12098-008-0211-6

[57] Camu F, Lauwers MH, Vanlersberghe C (1996) Side effects of NSAIDs and dosing recommendations for ketorolac. Acta Anaesthesiol Belg 47: 143–149. 8959200

[58] Tang BL (2011) Sirt1’s systemic protective roles and its promise as a target in antiaging medicine. Transl Res 157: 276–284. 21497775 10.1016/j.trsl.2010.11.006

[59] Satoh A, Stein L, Imai S (2012) The role of mammalian sirtuins in the regulation of metabolism, aging, and longevity. Handb Exp Pharmacol 206: 125–162. 10.1007/978-3-642-21631-2_7 PMC3745303 21879449

[60] Rothgiesser KM, Erener S, Waibel S, Luscher B, Hottiger MO (2010) SIRT2 regulates NF-kappaB dependent gene expression through deacetylation of p65 Lys310. J Cell Sci 123: 4251–4258. 21081649 10.1242/jcs.073783

[61] Kawahara TL, Michishita E, Adler AS, Damian M, Berber E, et al (2009) SIRT6 links histone H3 lysine 9 deacetylation to NF-kappaB-dependent gene expression and organismal life span. Cell 136: 62–74. 19135889 10.1016/j.cell.2008.10.052 PMC2757125

[62] Zhong L, D’Urso A, Toiber D, Sebastian C, Henry RE, et al (2010) The histone deacetylase Sirt6 regulates glucose homeostasis via Hif1alpha. Cell 140: 280–293. 20141841 10.1016/j.cell.2009.12.041 PMC2821045

[63] Zhang J, Lee SM, Shannon S, Gao B, Chen W, et al (2009) The type III histone deacetylase Sirt1 is essential for maintenance of T cell tolerance in mice. J Clin Invest 119: 3048–3058. 19729833 10.1172/JCI38902 PMC2752073

[64] Beier UH, Wang L, Bhatti TR, Liu Y, Han R, et al (2011) Sirtuin-1 targeting promotes Foxp3+ T-regulatory cell function and prolongs allograft survival. Mol Cell Biol 31: 1022–1029. 21199917 10.1128/MCB.01206-10 PMC3067815

[65] van der Meer AJ, Scicluna B, Lin J, Jacobson EW, Vlasuk GP, et al (2011) The first demonstration of clinical activity by a small molecule SIRT1 activator: SRT2104 reduces cytokine release and coagulation activation in a human endotoxemia model. Inflamm Res 60: S82.

