# Supplementary figures and images for: SIRT1 Activators Suppress Inflammatory Responses through Promotion of p65 Deacetylation and Inhibition of NF-κB Activity
Source: PLoS One. 2012 Sep 28;7(9):e46364. doi: 10.1371/journal.pone.0046364 (PMC3460821; doi:10.1371/journal.pone.0046364)

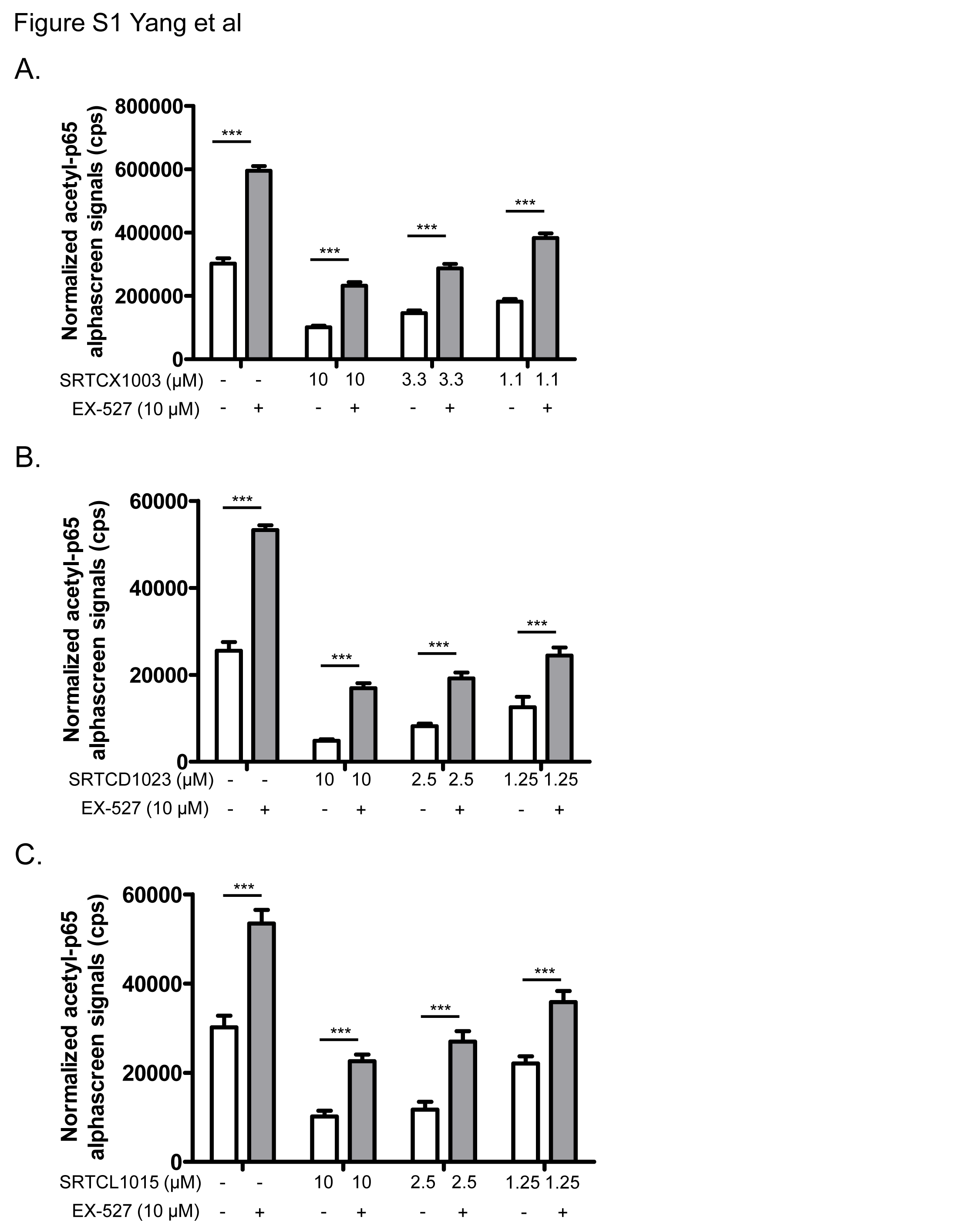

Supplement: Figure S1 — STACs promote SIRT1-mediated deacetylation of p65 protein. (A–C) Treatment of SRTCX1003, SRTCD1023 or SRTCL1015 at varied doses with or without 10 µM EX-527 on levels of acetylated p65 protein in U2OS cells. All error bars represent s.d. of at least 4 replicates. *P<0.05, **P<0.01 and ***P<0.001. (TIF) [file pone.0046364.s001.tif]

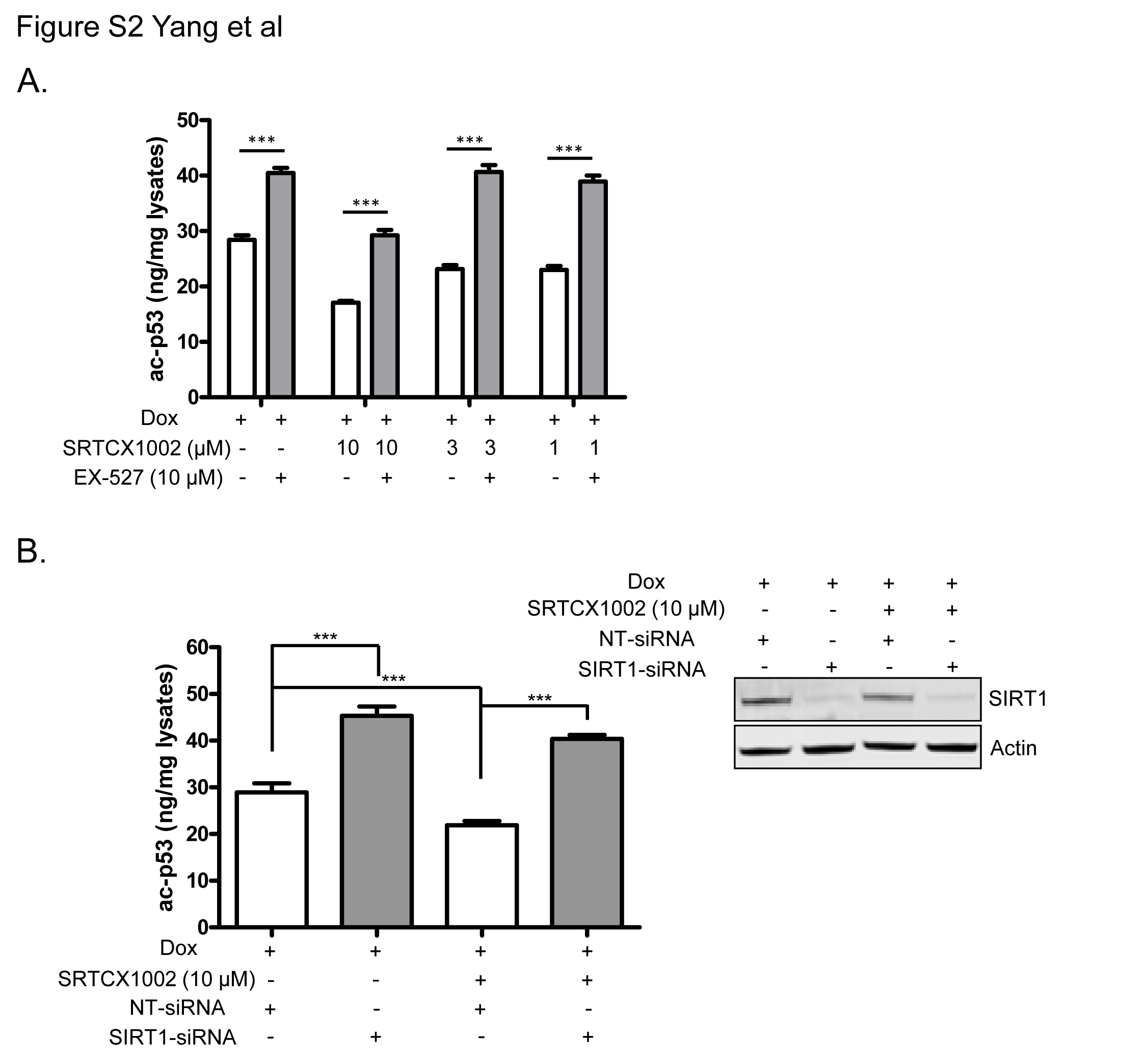

Supplement: Figure S2 — SRTCX1002 enhances SIRT1-mediated deacetylation of p53 protein. (A) Effects of varied concentrations of SRTCX1002 with or without 10 µM EX-527 on doxorubicin-induced p53 acetylation in U2OS cells. (B) Effects of SRTCX1002 on doxorubicin-induced p53 acetylation in U2OS cells transfected with either SIRT1-siRNA or NT-siRNA. Western blots in the right upper corner indicate that SIRT1 was knocked down by 70% by SIRT1-siRNA. All error bars represent s.d. of at least 4 replicates. *P<0.05, **P<0.01 and ***P<0.001. (TIF) [file pone.0046364.s002.tif]

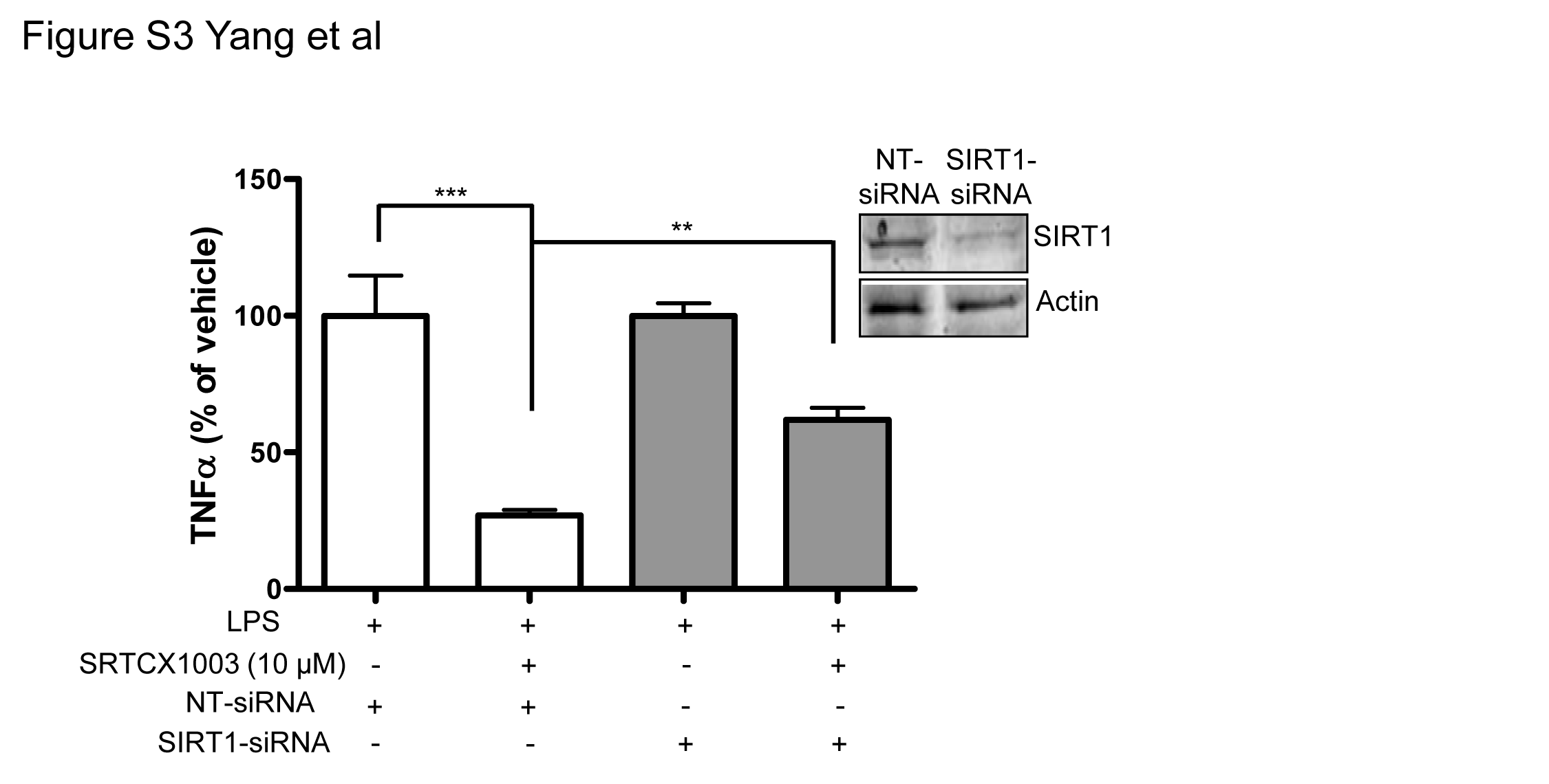

Supplement: Figure S3 — SRTCX1003 reduces LPS-induced TNFα secretion from RAW cells via SIRT1. Effects of SRTCX1003 on LPS-induced TNFα secretion from RAW cells transfected with either SIRT1-siRNA or NT-siRNA. Western blots in the right upper corner indicate that SIRT1 was knocked down by 70% by SIRT1-siRNA. All error bars represent s.d. of at least 3 replicates. *P<0.05, **P<0.01 and ***P<0.001. (TIF) [file pone.0046364.s003.tif]
