# Supplementary material for: SIRT1 Activators Suppress Inflammatory Responses through Promotion of p65 Deacetylation and Inhibition of NF-κB Activity
Source: PLoS One. 2012 Sep 28;7(9):e46364. doi: 10.1371/journal.pone.0046364 (PMC3460821; doi:10.1371/journal.pone.0046364)
Supplement: File S1 — Materials and Methods. (DOCX) [file pone.0046364.s004.docx]

**SUPPORTING INFORMATION S1**

**MATERIALS AND METHODS**

**SIRT1 biochemical assay**

Compounds that activated SIRT1 in the enzymatic assay on a p53-TAMRA peptide as described in our previous publication [1] were termed STACs. Activation potency was determined at 1/10 of the k_m_ values of peptide and NAD^+^, and tracked by the concentration of compound required to increase enzyme activity by 50% (EC1.5) and the percentage maximum activation achieved at highest doses of compound tested (Max Act (%)).

**Reference**

1. Milne JC, Lambert PD, Schenk S, Carney DP, Smith JJ, et al. (2007) Small molecule activators of SIRT1 as therapeutics for the treatment of type 2 diabetes. Nature 450: 712-716.
