# Supplementary material for: SIRT1 Activators Suppress Inflammatory Responses through Promotion of p65 Deacetylation and Inhibition of NF-κB Activity
Source: PLoS One. 2012 Sep 28;7(9):e46364. doi: 10.1371/journal.pone.0046364 (PMC3460821; doi:10.1371/journal.pone.0046364)
Supplement: Table S1 — STACs activate SIRT1 deacetylase activity in vitro. Table shows the EC1.5 and Max Act values of the STACs in SIRT1 enzymatic assay with p53-TAMRA peptide substrate and IC50 values of the STACs in the cellular p65 assay. (DOCX) [file pone.0046364.s005.docx]

Table S1

| **Compound ID** | **Biochemical assay** | | **Cellular p65 acetylation assay** |
| --- | --- | --- | --- |
|  | **EC1.5 (µM)** | **Max Act (%)** | **IC_50_ (µM)** |
| SRTCX1002 | 0.40 | 573 | 0.84 |
| SRTCX1003 | 0.61 | 328 | 1.42 |
| SRTCZ1001 | >50 | 112 | >25 |
| SRTCD1023 | 0.23 | 656 | 5.99 |
| SRTCL1015 | 0.08 | 775 | 0.46 |
| SRTCE1022 | 48.6 | 133 | >25 |
